# Research on a machine learning method for predicting discharge time of thyroid cancer patients receiving ^131^I treatment: a retrospective study

**DOI:** 10.3389/fphys.2025.1599657

**Published:** 2025-09-26

**Authors:** Chen Zhang, Dandan Zhang, Lijun Tang, Feng Tian

**Affiliations:** ^1^ Department of Nuclear Medicine, Jiangsu Province Hospital, The First Affiliated Hospital with Nanjing Medical University, Nanjing, China; ^2^ Department of Operations Management, Jiangsu Province Hospital, The First Affiliated Hospital with Nanjing Medical University, Nanjing, China; ^3^ China Hospital Reform and Development Research Institute of Nanjing University, Nanjing Drum Tower Hospital, Nanjing, China

**Keywords:** ^131^I, thyroid cancer, effective half-life, support vector machine, discharge time

## Abstract

Radioactive iodine-131 (^131^I) based internal irradiation therapy has become one of the main methods for treating thyroid cancer, but patient usually need to be hospitalized after taking ^131^I until the residual activity meets the discharge criteria. However, the complex metabolism of ^131^I drug in individualized patient may make it difficult to assess when patients would meet discharge criteria, thereby increasing the hospital stay. In this study, some basic data of 1,044 thyroid cancer patients received ^131^I treatment at the First Affiliated Hospital with Nanjing Medical University from January 2022 to January 2024 are collected. Numerical analysis methods are used to analyze the absorption and metabolism of ^131^I drug in different patients and support vector machine (SVM) model is used to predict the discharge time of different patients. Results show that the effective half-life of ^131^I in both male and female patients are 10.35 h and 9.64 h, whose residual activity less than 400 MBq after 48 h of taking ^131^I. While the effective half-life of ^131^I in both male and female patients are 14.07 and 13.47 h for that the residual activity are greater than 400 MBq after 48 h of taking ^131^I. Furthermore, a discharge time prediction method based on SVM has been developed and the accuracy and precision of this method in predicting whether a patient could be discharged from the hospital after 48 h of taking the ^131^I drug are 88.04% and 96.89%. These results show that the discharge time prediction method could be expected to improve the rotation efficiency of nuclear medicine wards and provide timely treatment for more thyroid cancer patients receiving ^131^I treatment in the future.

## Introduction

According to the GLOBOCAN 2022 database of cancer incidence and mortality by the WHO International Agency for Research on Cancer, thyroid cancer is ranking in seventh place for incidence (Bray et al., 2024). Thyroid cancer can be treated through surgical removal of the thyroid gland (Nguyen et al., 2015). However, some effects would have an impact on the choice of surgery, such as the size and grading of tumors et al. Studies have shown that the thyroid cancer may recur when surgical resection is incomplete (CSJGs, 2015; Sippel and Chen, 2009). Moreover, the patient’s quality of life after surgery of thyroid gland may also be affected (Dogan et al., 2017; Singer et al., 2012). Recent years, due to the high uptake of iodine by the thyroid gland, the utilization of radioactive iodine-131 drugs (i.e., [^131^I]NaI) has become one of the main methods for the treatment of thyroid cancer (Verburg et al., 2020; Giovanella et al., 2023).

Since the maximum energy of the *β* ^*-*^ ray released by ^131^I is only 606.5 keV, ^131^I could only deposit energy in its accumulated tissue, ultimately achieving the killing of tumor cells (Al-jubeh et al., 2012; Kim et al., 2019). Considering the physical half-life of ^131^I (i.e., 8.02 d) and the dose deposited in tumor areas, the activity typically used to treat thyroid cancer ranges from 100 to 200 mCi in clinical (Gao et al., 2024; Tran et al., 2022; Nguyen et al., 2024). As a result of the thyroid cancer patients received the ^131^I treatment may have a radiation dose impact on the public, and different countries and regions have set some discharge criteria to protect the public (Venencia et al., 2002; Tunçel et al., 2016). For example, in Argentina, the Nuclear Regulatory Authority has stipulated that the patient received ^131^I treatment needed to be hospitalized in special wards for 2 or 3 d if patients receive a dose >1 GBq (30 mCi), or if the emitting radiation dose rate is >50 μSv/h at 1 m. The US Nuclear Regulatory Commission regulatory guide (No. 8.39) allows the release of differentiated thyroid carcinomas patients based on a measured dose rate of 7 mR/h at 1 m (Shahhosseini et al., 2004). According to the GB 18871-2002 and GBZ 120-2020 formulated by relevant departments in China, patients can be discharged from the hospital when the residual activity less than 400 MBq (Zhou et al., 2019; Jin et al., 2018).

When a thyroid cancer patient is admitted to the nuclear medicine departments, doctors would mainly analyze the patient’s condition to determine the administration activities of ^131^I. However, it is difficult to estimate when the patient would meet the discharge criteria after taking the ^131^I. Zahra, et al. studied the radiation exposure rate of 100 patients who were treated with 3.7, 5.5 or 7.4 GBq of ^131^I, the exposure rates after each of the three first days of hospitalization were 30, 50 and 70 μSv/h at 1 m, and all patients had an acceptable dose rate on days 2 and 3 that allowed their hospital discharge (Azizmohammadi et al., 2013). Sometimes, patients could meet the discharge criteria after 48 h of taking ^131^I drug, but due to the complex absorption or metabolic abilities of ^131^I drug in patients, residual activity of ^131^I drug in some patients may exceed the discharge criteria after 48 h. In order to ensure the radiation safety, the hospitalization duration for different thyroid cancer patients is often fixed, resulting in some patients being unable to leave the hospital even after meeting the discharge criteria. Such behavior not only wastes medical resources and leads to some patients being unable to receive timely treatment to some extent, but also imposes unnecessary financial burdens on them. Considering the reality that the incidence of thyroid cancer in China has been increasing year by year in recent years, more and more patients would receive ^131^I treatment. However, the inability to accurately assess patient’s discharge time in advance has led to a waste of medical resources, making it challenging to ensure patients’ safety.

If the discharge time for patients receiving ^131^I treatment could be accurately assessed before or during their hospitalization, it would not only curtail their hospital stay and waiting period but also enhance the rotation efficiency of nuclear medicine wards. To achieve this goal, the new methods and technologies should be established. In recent years, artificial intelligence has made great progress in all fields of medicine. Related researches on disease diagnosis using patient’s data combined with artificial intelligence has provided methods and ideas for solving the problem of predicting patient discharge time in the real world (Iqbal et al., 2021; Iqbal et al., 2022). In this study, the metabolism of [^131^I] NaI in different patients are analyzed, and a new technology for accurately predicting the discharge time of patient receiving ^131^I treatment based on the basic data of patients, the metabolism of ^131^I drug and machine learning algorithm is established and the performance are evaluated accordingly.

## Materials and methods

### Basic information of thyroid cancer patients selected in this study

In this study, some data of 1,044 thyroid cancer patients who received ^131^I treatment at the department of nuclear medicine of the First Affiliated Hospital with Nanjing Medical University from January 2022 to January 2024 are collected. The basic information of these patients could be found in Table 1, which consisted of number of cases, gender, age, ^131^I drug activity used.

**TABLE 1:** Basic information of thyroid cancer patients selected in this work.

Gender	Average age (years)	Median age (years)	Number of patients taking 100 mCi ^131^I	Number of patients taking 150 mCi ^131^I	Number of patients taking 200 mCi ^131^I
Male	41.67 ± 12.72	39	47	345	16
Female	42.55 ± 12.83	42	106	507	23

### Method and equipment for monitoring the residual activity of patients

After taking a sufficient amount activity of ^131^I drug orally at one time, the patient returned to the ward to wait for the ^131^I drug to be metabolized in the body until the residual activity in the body met the discharge criteria. During hospitalization, patients undergo residual activity monitoring every 24 h after taking ^131^I drug in a specific dose monitoring room. A real-time dosimeter is placed on the wall of the monitoring room, which produced by Shanghai Juyin Technology Co., Ltd. The basic information of the radiation monitoring system are shown in Table 2. Through calibration, the dosimeter can obtain the radiation activity of ^131^I through the dose rate measured. The patient is required to stand on the identification line for 1 min which is 1 m away from the dosimeter. The real-time reading of the dosimeter will be displayed on the remote monitoring device of the nurse station, and the staff will record the reading of the dosimeter when it is stable. By analyzing the residual activity of ^131^I drug in different patients at a specific time after taking the medicine, the metabolisms of ^131^I drug in different patients can be revealed.

**TABLE 2:** The basic information of radiation monitoring system.

Parameter	Value
Dose range	0.1 μSv/h ∼ 10 mSv/h
Energy range	33 keV ∼ 3 MeV
Sensitivity	∼2.2 cps/μSv/h(@137Cs)
Dose rate linearity	≤ ±10%(0.1 μSv/h ∼ 10 mSv/h)

### Machine learning algorithms and evaluation parameters used in this study

In order to achieve more efficient rotation and utilization of nuclear medicine wards and ensure timely discharge of patients, machine learning algorithms could be used. Basing the previous comparison of the performance of different machine learning algorithms, a support vector machine (SVM) algorithm is used to analyze the relationship between when the patient can discharge and collected patients’ data which consisted of gender, age, ^131^I drug activity used, and the metabolism of ^131^I drug (Suthaharan and SJMlm, 2016; WSJNb, 2006). The regularization parameter of SVM is set to 1. Patients with residual activity less than 400 MBq at 48 h after taking ^131^I drug are classified into one category (MC), while patients with residual activity greater than 400 MBq are classified into another category (NMC). 80% of patients are selected randomly as training data and rest 20% of patients as testing data. SMOTE is used to train and optimize the SVM model, and stratified sampling is adopted during train-test splitting to preserve the original class proportions in both training and testing sets. All the training and optimization involved in this study were done basing Python 3.11.3 (Chao et al., 2014; Markowetz et al., 2003). And quantitative parameters such as accuracy, precision, sensitivity, specificity, F1-score, AUC value are used to analyze the test results (Abjijoml, 2013).

## Results

### ^131^I activity changes in thyroid cancer patients

**FIGURE 1:**
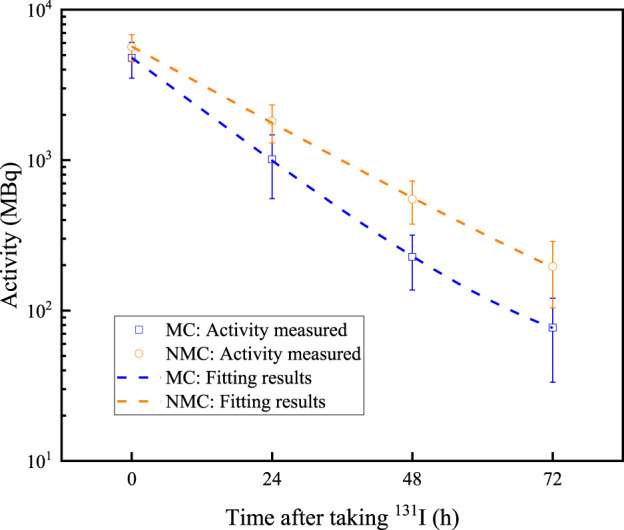
Residual activity decreases over time in male thyroid cancer patients. Dots represent the measured data, and the dashed lines represent the fitting results.

**FIGURE 2:**
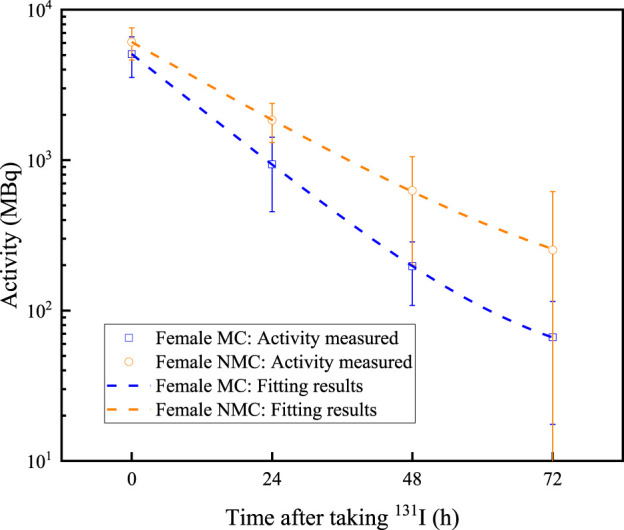
Residual activity decreases over time in female thyroid cancer patients. Dots represent the measured data, and the dashed lines represent the fitting results.

For all patients selected in this study, NMC patient accounts for 16.08% of the total patients. NMC male patient accounts for 8.51% of the total patients, and NMC male patient accounts for 22.34% of all male patients. NMC female patient accounts for 7.57% of the total patients, while accounts for 12.23% of all female patients. The residual activity changes in all thyroid cancer patients are shown in Figures 1, 2. The value of residual activity measured are fitted using the e-exponential function to obtain the effective half-life of ^131^I in different patients. The fitting results of male MC, male NMC, female MC and female NMC are shown in Formula 1–4. And the fitted values and measured values of the residual activity of ^131^I in patients after taking the ^131^I drug for a specific time are shown in Table 3. From these results, it can be seen that the residual activity attenuation of ^131^I drug in all patients follows the exponential attenuation law. The effective and biological half-life of ^131^I for different patients are calculated according to the measured results are shown in Table 4.

$$\text{Male MC }\;Activity=4761.04\;\cdot \;e^{-\frac{t}{14.93}}+38.23\left(R^{2}=99.987\%\right)$$ (1)

$$\begin{array}{cc} \text{Male NM}C & Activity=5665.07\cdot e^{-\frac{t}{20.30}}+30.77\left(R^{2}=99.955\%\right) \end{array}$$ (2)

$$\text{FemaleMC }\;Activity=5033.50\cdot \;e^{-\frac{t}{13.91}}+37.74\left(R^{2}=99.999\%\right)$$ (3)

$$\begin{array}{cc} \text{Female NM}C & Activity=5987.29\cdot e^{-\frac{t}{19.44}}+109.73\left(R^{2}=99.995\%\right) \end{array}$$ (4)

**TABLE 3:** The residual activity values in patient at different time points.

Category		0 h	24 h	48 h	72 h
Male MC	Measured value	4,780.65 (4,630.59, 4,930.70)	1,012.00 (958.39, 1,065.61)	227.37 (216.76, 237.97)	77.13 (72.03, 82.22)
	Fitted value	4,490.82	992.28	229.41	76.54
Female MC	Measured value	5,058.57 (4,926.63, 5,190.51)	937.24 (895.42, 979.06)	196.63 (188.97, 204.30)	66.14 (61.92, 70.36)
	Fitted value	4,722.08	934.24	197.41	66.18
Male NMC	Measured value	5,663.81 (5,409.41, 5,918.21)	1814.37 (1701.86, 1926.88)	550.36 (511.75, 588.97)	196.07 (175.87, 216.26)
	Fitted value	5,423.53	1767.59	563.25	194.02
Female NMC	Measured value	6,009.06 (5,661.10, 6,357.01)	1833.54 (1703.37, 1963.71)	619.63 (514.98, 724.29)	247.11 (157.21, 337.02)
	Fitted value	5,796.82	1851.79	616.61	257.21

(): The 95% confidence interval of the data.

**TABLE 4:** The effective and biological half-life of ^131^I for different patients.

Category	Average age (years)	Effective half-life (h)	Biological half-life (h)
Male MC	41.84 ± 14.51	10.35	10.94
Female MC	43.33 ± 16.42	9.64	10.15
Male NMC	42.17 ± 13.88	14.07	15.18
Female NMC	52.10 ± 33.59	13.47	14.49

### Iodine uptake rate in different thyroid cancer patients

**FIGURE 3:**
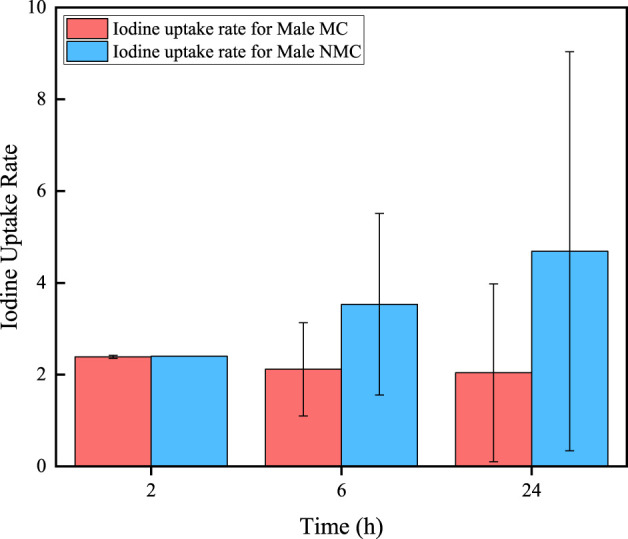
Iodine uptake rate in different time for male thyroid cancer patients.

**FIGURE 4:**
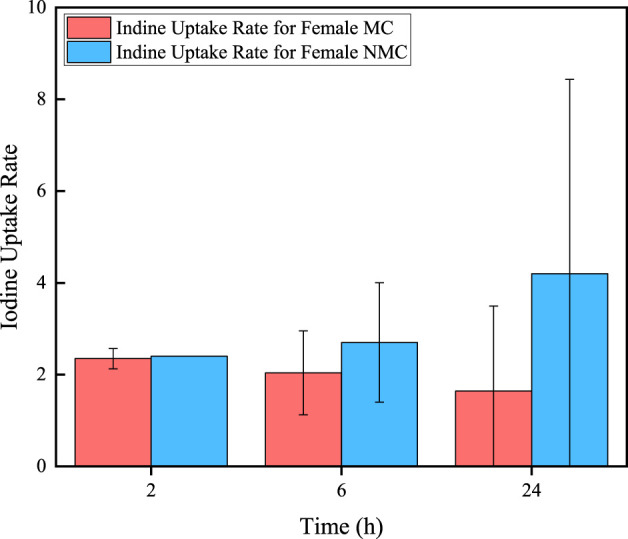
Iodine uptake rate in different time for female thyroid cancer patients.

In addition, 70 of all thyroid cancer patients would take iodine uptake rates test after 2, 6, and 24 h of taking ^131^I drug (male/NMC: 26/9, female/NMC: 44/5). The results are shown in Figures 3, 4. It can be seen that for MC patients, their iodine uptake rate shows a downward trend, while for NMC patients, it shows a upward trend.

### Discharge time prediction of thyroid cancer patients based on support vector machine

**FIGURE 5:**
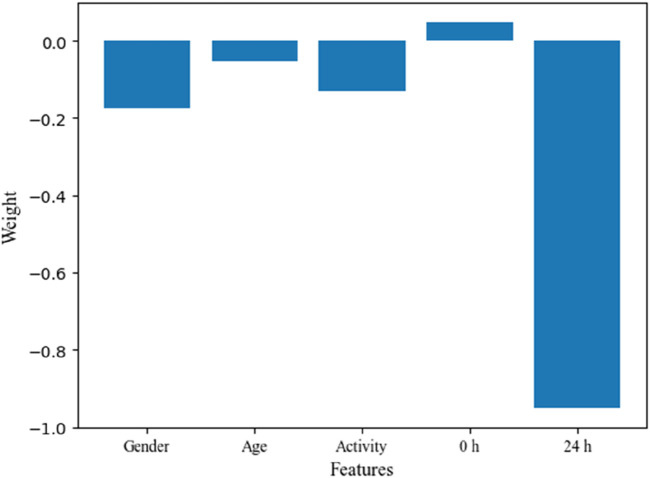
The weight of different features of patient about discharge judgment.

**FIGURE 6:**
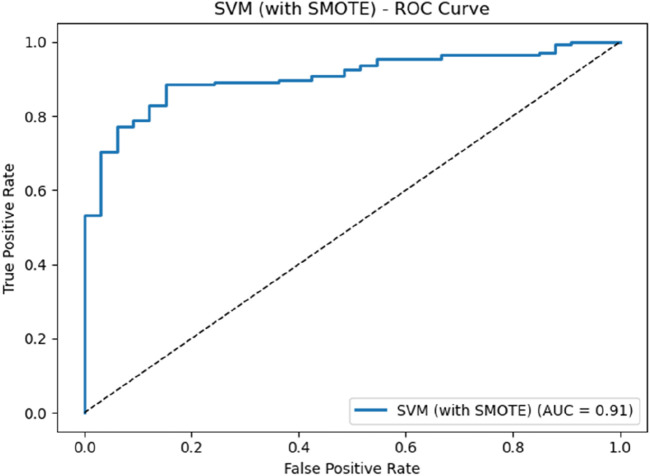
ROC curve for predicting whether patient can be discharged after 48 h of taking ^131^I.

There are significant individualized differences in the absorption and metabolism of ^131^I drug among thyroid cancer patients, which also would affect the rotation and utilization of nuclear medicine ward to some extent. Based on the patient information collected, and the metabolisms of ^131^I drug, the impact weights of patient information on the classification of MC and NMC are analyzed through feature engineering, and the results are shown in Figure 5. From the results, it can be seen that for the patient information collected in this study, the patient’s gender and the activity after 24 h of taking ^131^I drug are key factors affecting whether the patient could meet the discharge criteria after 48 h of taking ^131^I drug. Finally, based on the data collected, the SVM model is used to predict whether patients could meet the discharge criteria after 48 h of taking the ^131^I drug. The ROC curve and the value of quantitative parameters are shown in Figure 6 and Table 5, respectively. It can be seen that the ROC curve with an AUC of 0.92, which demonstrates that the SVM classifier has good discrimination ability. Combined with the accuracy of 88.04%, precision of 96.89%, sensitivity of 88.64%, and an F1-score of 0.93, the SVM model could effectively identify true positives while maintaining an acceptable false positive rate. These results show that based on the SVM algorithm and the metabolism of ^131^I drug of individual patent collected, whether the patient could be discharged from the hospital after 48 h of taking ^131^I can be accurately predicted.

**TABLE 5:** The value of quantitative evaluation parameters.

Parameter	Accuracy	Precision	Sensitivity	Specificity	F1-score	AUC
Value	88.04%	96.89%	88.64%	84.85%	0.93	0.91

## Discussion

In recent years, the incidence of thyroid cancer has continued to increase, partly due to the increased incidence of diseases and also partly due to the improvement of diagnostic techniques. ^131^I drug has become an important technology for the treatment of thyroid cancer. However, due to the presence of residual radioactivity in patients after taking the ^131^I drug, patients generally required hospitalization until their residual activity meet the discharge criteria. Considering the large number of thyroid cancer patients and the shortage of medical resources, in order to ensure treatment efficiency and the radiation safety of the staff and public, it is necessary to clarify the patient’s discharge status in advance to ensure the rotation of the nuclear medicine wards.

Some data of 1,044 thyroid cancer patients received ^131^I treatment at the department of nuclear medicine of the First Affiliated Hospital with Nanjing Medical University from January 2022 to January 2024 are collected. Firstly, the metabolisms of ^131^I drug of different patients are analyzed. The data shows that approximately 83.92% of patients met the discharge criteria, which the residual activity less than 400 MBq after 48 h of taking ^131^I. The discharge rate of female patients is 87.77%, while that of male patients is only 77.66%. This may be due to the number of male patients collected in this study is much smaller than female patients, resulting in significant statistical errors. And it is also possible that male thyroid cancer patients have poorer metabolic capacity for ^131^I than female patients. The residual activity in patient decreases exponentially with the time after taking ^131^I drug. By fitting the activity profiles of MC and NMC patients, the effective half-life of ^131^I in both male and female MC patients are 10.35 h and 9.64 h, while the effective half-life of male and female NMC patients are 14.07 h and 13.47 h, respectively. The longer biological half-life would be mainly due to delayed renal excretion of ^131^I. In addition, these data further indicate that the male patients have a longer effective half-life of ^131^I than female patients both of MC and NMC patients. In addition, there are also differences in the changes in iodine uptake rate in different patients. For NMC patients, the iodine uptake rate in 2 h, 6 h and 24 h after taking ^131^I drug showed an upward trend, while in MC patients showed a downward trend. Iodine uptake rate can also provide a reference for assessing when a patient can be discharged to some extent.

In order to accurately determine whether patients could be discharged after 48 h of taking ^131^I, a machine learning prediction method based on SVM has been developed with patient data collected and the metabolisms of ^131^I drug. The results show that the accuracy and precision of this method to determine whether a patient is MC or NMC are 88.04% and 96.89%, respectively. Considering that 83.92% of all patients are MC, these results indicate that the discharge time prediction method based on the metabolisms of ^131^I drug and SVM established in this work can improve the accuracy of predicting patient discharge time, thereby providing technical support for the rotation and utilization of nuclear medicine wards to some extent. However, the patient information currently collected in this work is relatively limited, and more information of patient, such as tumor staging, would be collected, and combined with the residual activity at more time points after taking the ^131^I drug, so as to achieve a rough assessment of the patient’s discharge time before the patient is hospitalized in the future. In addition, this study is currently a single-center study, and multi-center studies would be conducted in the future to explore the impact of more factors in the real-world on the performance of discharge time prediction method, so as to optimize the method to help more patients and more centers.

## Conclusion

The number of new cases of thyroid cancer has been continuously increasing year by year, and ^131^I drug has become an important technology for the treatment of thyroid cancer. However, due to individual differences in the absorption of ^131^I drug by patients, there are also individual differences in when patients meet the discharge criteria after taking the ^131^I drug. A discharge time prediction method basing the metabolisms of ^131^I drug in different patients and the SVM algorithm is established in this study, and the results show that this method could be expected to improve the rotation efficiency of nuclear medicine wards and provide timely treatment for more patients in the future.

## Data Availability

The raw data supporting the conclusions of this article will be made available by the authors, without undue reservation.

## Abbreviations

^131^I, Radioactive iodine-131; SVM, Support vector machine; MC, Meet discharge conditions; NMC, Not meet discharge conditions.
